# The Mouse Blood-Brain Barrier Transcriptome: A New Resource for Understanding the Development and Function of Brain Endothelial Cells

**DOI:** 10.1371/journal.pone.0013741

**Published:** 2010-10-29

**Authors:** Richard Daneman, Lu Zhou, Dritan Agalliu, John D. Cahoy, Amit Kaushal, Ben A. Barres

**Affiliations:** 1 Department of Anatomy, University of California San Francisco, San Francisco, California, United States of America; 2 Department of Neurobiology, Stanford University School of Medicine, Stanford, California, United States of America; 3 Department of Developmental Biology, Stanford University School of Medicine, Stanford, California, United States of America; 4 Department of Biomedical Informatics, Stanford University School of Medicine, Stanford, California, United States of America; Boston University School of Medicine, United States of America

## Abstract

The blood-brain barrier (BBB) maintains brain homeostasis and limits the entry of toxins and pathogens into the brain. Despite its importance, little is known about the molecular mechanisms regulating the development and function of this crucial barrier. In this study we have developed methods to highly purify and gene profile endothelial cells from different tissues, and by comparing the transcriptional profile of brain endothelial cells with those purified from the liver and lung, we have generated a comprehensive resource of transcripts that are enriched in the BBB forming endothelial cells of the brain. Through this comparison we have identified novel tight junction proteins, transporters, metabolic enzymes, signaling components, and unknown transcripts whose expression is enriched in central nervous system (CNS) endothelial cells. This analysis has identified that RXRalpha signaling cascade is specifically enriched at the BBB, implicating this pathway in regulating this vital barrier. This dataset provides a resource for understanding CNS endothelial cells and their interaction with neural and hematogenous cells.

## Introduction

The vasculature of the CNS forms an endothelial barrier, not found in other tissues, that limits the flow of molecules and ions between the blood and the brain. This BBB is crucial for maintaining brain homeostasis and for limiting the penetration of toxins and pathogens into the brain [1]. The importance of the BBB is highlighted by the severe pathology of diseases in which it is disrupted, including stroke, edema, brain traumas and multiple sclerosis [2]. The BBB also poses an obstacle for the treatment of neurological disorders as it can greatly limit drug delivery into the brain [3].

Many of the properties of the BBB are manifested in the endothelial cells which make up the walls of the blood vessels. Endothelial cells in the brain differ from endothelial cells in other tissues in that they are held together by high electrical resistance tight junctions and contain few transcytotic vesicles, limiting the paracellular and transcellular flow of molecules from the blood into the brain [4]. In addition, CNS endothelial cells express a variety of transporters, both to provide the CNS with specific nutrients, and also to efflux potential toxins from the CNS. Whereas the endothelial cells form the barrier, transplantation studies have demonstrated that these properties are not intrinsic to the endothelial cells but signaled by the brain microenvironment [5]. Evidence suggests that the BBB is induced and regulated by its close association with astrocytes, pericytes and other neural cells, however the molecular nature of these interactions remains a mystery [6], [7], [8].

Several studies have taken different approaches to understand the genomics of the BBB, including microarray analysis, subtractive hybridization and serial analysis of gene expression [9], [10], [11]. These studies have provided information about the gene expression of CNS vessels but had two limitations. First, because these studies used whole vessel fractions, which contain many different cell types, the transcriptomes of the individual cellular components, the endothelial cells and the pericytes, are not yet known. Second, these studies compared the gene expression of brain vessels with whole tissue fractions instead of purified peripheral vessels. Paramount to understanding the function of the BBB is a comparison of purified endothelial cells from brain with purified endothelial cells from peripheral tissues.

Here we utilize fluorescence-activated cell sorting (FACS) to highly purify endothelial cells from the brain, liver and lung of Tie2GFP transgenic mice [12]. Using Affymetrix microarrays to gene profile these purified cell populations, we have identified a set of genes whose expression is highly enriched at the BBB. These genes include tight junction molecules, transporters, signaling molecules, and other molecule classes. This analysis has implicated Wnt and RXRalpha signaling cascades in regulating this crucial barrier.

## Materials and Methods

### Ethics Statement

All experiments were approved by Stanford University IACUC committee, approved protocol #10726.

### FACS purification of GFP^+^ cells

Homozygous Tie2GFP mice (strain 003658) were obtained from Jackson labs and bred to maintain homozygosity. Cell suspensions were prepared from cerebral cortex, liver or lung based on procedures previously described [13], [14]. For brain samples, the cerebral cortex was dissected away from the forebrain, then the meninges were peeled off with fine forceps. For liver samples, peripheral regions of each lobe were utilized to avoid the hepatic portal vein ensuring the tissue vasculature consisted primarily of capillaries. Whole lung lobes were utilized. Each tissue was diced with a scalpel, and enzymatically dissociated with a papain solution (40 U/ml Worthington-3126) containing L-cysteine (0.4 mg/ml, Sigma C 7477) and DNase (125 U/ml, Worthington LS002007) for 1.5 hours at 35°C, prior to mechanical trituration in a solution containing ovomucoid (2 mg/ml, Roche 109878), DNase (125 U/ml) and BSA (1 mg/ml, Sigma A8806), to yield a cell suspension, which was recovered by centrifugation. Cell suspensions were re-suspended in FACS buffer (DPBS, 0.02%BSA with propidium iodide), and endothelial cells were FACS purified based on GFP fluorescence utilizing a FACS Vantage SE sorter (Becton Dickinson) and CellQuest software. For each tissue, background GFP fluorescence was determined by FACS analysis of cell suspensions from wild type FvB mice (Charles River), and dead cells were eliminated by high propidium iodide fluorescence. Forward scatter and side scatter analysis were also used as gates to limit the sorting to single live cells. For additional pericyte/VSM depletion, cell suspensions were stained with a goat anti-mouse PDGFRbeta primary antibody (R & D systems), and a donkey anti-goat secondary antibody labeled with APC. For these experiments, background APC fluorescence was gated with cell suspensions incubated with secondary, but not primary antibody. In each case, two rounds of sorting were performed for maximal purity, based on reanalysis. Cortical GFP^−^ cells were isolated from Tie2GFP^+^ mice with gating based on background GFP fluorescence from wild type FvB mice.

### GeneChip Analysis

Purification of RNA, generation of biotinylated cRNA, subsequent hybridization to Affymetrix Mouse Genome 430 2.0 Arrays and raw image analysis with Affymetrix GCOS 1.3 software was performed as previously described [14]. Three biological replicates were used for each analysis of adult cells, whereas five biological replicates were utilized for postnatal samples. Each biological replicate represents a purification from different mice performed on different days. The Significance Analysis of Microarrays (SAM) method was used to determine genes that were significantly different between cell populations. For this analysis, samples were grouped into 6 groups: brain parenchyma (GFP^−^), brain vascular (GFP^+^), brain endothelial (GFP^+^PDGFRbeta^−^), postnatal brain GFP^+^, liver endothelial and lung endothelial. Probe sets were included in this statistical analysis if they were defined as being called present and having an intensity level greater than 200 in at least two-thirds of the samples in at least one group. The significance thresholds were set for a false discovery rate of less than 1% with a fold-change of greater than 2.0×. For each data analysis presented in this paper, fold-enrichment was calculated as expression value of one data set divided by the second. In each table presented, probe sets were included only if gene expression was ≥200 in the enriched data set. In comparisons of brain endothelial to lung and liver endothelial samples, or comparisons of adult and postnatal brain samples, GFP^+^ samples for each tissue were compared, and CNS pericyte/VSM enriched transcripts (brain GFP^+^/GFP^+^PDGFRbeta^−^>2) were excluded. To identify signaling and metabolic pathways enriched in specific data sets, Ingenuity Pathway Analysis software core analysis (Ingenuity systems) was utilized to determine the statistical significance of enrichment for the pathways within a given data set.

### *In situ* Hybridization

*In situ* hybridization on P20 fresh frozen mouse brain and liver sections was performed as described [15] with a few modifications, omitting the treatment with Proteinase K. The antisense mRNA probes were generated using the DIG RNA Labeling Kit (Roche Applied Science, Indianapolis, IN). Ten genes were analyzed by *in situ* hybridization on P20 fresh frozen mouse brain and liver sections: Pecam (*Sma*I, T7), Cldn5 (*Xho*I, T3), Apcdd1 (*HindIII*, T7), Abcb1a (*EcoR*V, T7), Slco1c1 (*Xho*I, T3), Slco1a4 (*XhoI*, T3), Slc22a8 (*Xho*I, T3), Slc7a5 (*SalI*, T3), Itih5 (*Sal*I, T3), Foxq1 (*EcoR*V, T7). Apcdd1 cDNA was amplified by RT-PCR using total RNA isolated from FACS isolated Tie2GFP P60 brain endothelial cells. The other full length or partial cDNAs were obtained as mouse ESTs from Open Biosystems (Hunstville Al).

## Results

### Purification of CNS Vascular Cells from Tie2GFP Mice

**Figure 1:**
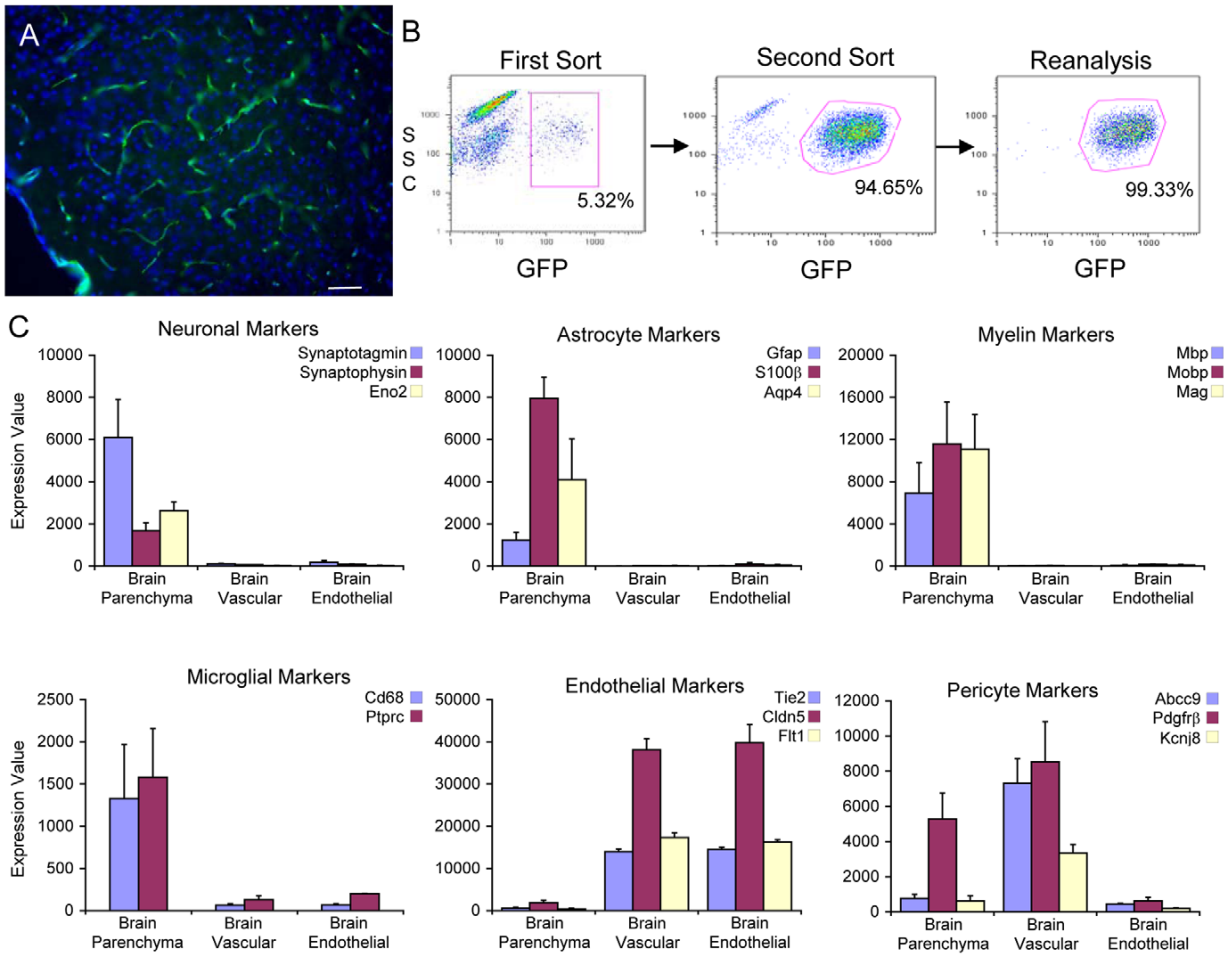
Purification of endothelial cells from the cerebral cortex of Tie2GFP mice. A) GFP is expressed in the vasculature of Tie2GFP transgenic mice. GFP (green) can be visualized in a vascular pattern in tissue cryosections of cerebral cortex of an adult Tie2GFP transgenic mouse. Nuclei were stained with DAPI (blue). Bar, 50 microns. B) FACS purification of GFP^+^ cells from the cerebral cortex of Tie2GFP mice. Single cell cortical suspensions were sorted for GFP fluorescence twice, isolating a GFP^+^ cell population that is greater than 99.3% pure. Y-axis represents side scatter co-efficient, X-axis represents GFP fluorescence, numerical values represent percent of total cells sorted that are within gated region. C) Purity of FACS sorted cells. GeneChip transcriptional profiling of GFP^−^ (parenchyma), GFP^+^ (vascular) and GFP^+^PDGFRbeta^−^ (endothelial) fractions from the cerebral cortex of Tie2GFP mice. Neuronal, astrocyte, oligodendrocyte, microglial, endothelial and pericyte markers were analyzed to detect the purity of sample for each FACS sorted sample. The GFP^+^ sample contains a mixture of endothelial cells and pericytes, such that an additional PDGFRbeta negative selection is needed to generate a pure endothelial cell population. Error Bars represent standard error of the mean (SEM).

To purify cells from the vasculature of different tissues we took advantage of Tie2GFP transgenic mice available from Jackson labs. These mice express the green fluoresecent protein (GFP) under the pan-endothelial Tie2 promotor, and GFP fluorescence can be visualized in the vasculature of the brain, liver, lung, spleen, kidney, muscle and other tissue cryosections (Figure 1a)[12]. When single cell suspensions of adult cerebral cortex were analyzed by FACS, approximately 5% of the cells were GFP^+^, a figure which matches the proportion of vascular cells in the brain (Figure 1b)[12]. Using a double FACS procedure to ensure the highest purity (Figure 1b) we purified three different cell populations: GFP^−^ (parenchymal), GFP^+^ (vascular cell), and GFP^+^PDGFRbeta^−^ (endothelial cell). For the latter cell population an anti-PDGFRbeta depletion step was utilized to deplete pericytes/vascular smooth muscle cells (VSMs) from the sample.

The purity of these cell populations was ascertained by using Affymetrix microarrays to compare the relative mRNA levels of specific markers for neurons (synaptotagmin I, synaptophysin, neuronal enolase), astrocytes (GFAP, Aq4, s100beta), oligodendrocyte lineage cells (MBP, MAG, MOBP), microglial cells (cd68, Ptprc), endothelial cells (Tie2, claudin 5, and Flt1) and pericytes/VSMs (PDGFRbeta, Abcc9, and Kcnj8) (Figure 1c). As expected, the neuronal and glial markers were all present in the brain parenchyma sample and low to absent in the vascular and endothelial cell samples, indicating these samples did not contain neural cells. The vascular cell sample contained endothelial and pericyte/VSM cell markers indicating it was a mixture of these two cell populations, whereas the endothelial cell sample contained endothelial cell markers but not pericyte/VSM markers. This separation allowed us to purify endothelial cells and identify the endothelial cell transcriptome. Furthermore, comparing the vascular and endothelial cell data sets we are able to identify many genes that are highly enriched to pericytes/VSM. A master data table of all data sets described can be found in Table S1.

In Table S2, we list the enriched CNS endothelial genes, defined as those GeneChip probe sets significantly enriched greater than two-fold in the endothelial:parenchymal fractions (GFP^+^ PDGFRbeta^−^:GFP^−^). We further present a list of the pericyte/VSM enriched genes, as defined as enriched greater than two-fold in the Vascular:Endothelial (GFP^+^:GFP^+^PDGFRbeta^−^) fractions (Table S3). In Table 1 we list the most endothelial and pericyte enriched probe sets which provide many new endothelial and pericyte/VSM specific markers within the brain.

**Table 1:** Most enriched CNS endothelial and pericyte transcripts.

A.Probe Set ID	Gene Symbol	Brain Endothelial/Parenchyma	B.Probe Set ID	Gene Symbol	Vascular/Endothelial
1421078_at	Tcf23	96.1	1423341_at	Cspg4	40.9
1438558_x_at	Foxq1	71.9	1418773_at	Fads3	38.8
1422905_s_at	Fmo2	62.1	1455098_a_at	Vtn	32.7
1460371_at	Hspa12b	62.1	1416286_at	Rgs4	27.8
1438227_at	She	59.4	1437902_s_at	Rarres2	27.1
1436939_at	Unc45b	55.4	1459713_s_at	Tmem16a	25.8
1419467_at	Clec14a	52.4	1425103_at	Ace2	24.6
1448117_at	Kitl	52.3	1433525_at	Ednra	24.3
1447933_at	Kif26a	52.0	1426285_at	Lama2	22.2
1438325_at	Evi1	51.3	1434141_at	Gucy1a3	22.0
1422966_a_at	Tfrc	48.3	1417439_at	Cd248	21.9
1416564_at	Sox7	48.0	1448649_at	Enpep	21.6
1443832_s_at	Sdpr	47.9	1417148_at	Pdgfrb	20.1
1438532_at	Hmcn1	47.7	1438658_a_at	Edg3	20.0
1449184_at	Pglyrp1	47.6	1436411_at	Atp13a5	20.0
1452352_at	Ctla2b	44.8	1438751_at	Slc30a10	19.6
1435701_at	Cyyr1	44.8	1427221_at	Xtrp3s1	18.9
1436419_a_at	1700097N02Rik	44.2	1448421_s_at	Aspn	18.2
1449328_at	Ly75	42.0	1418142_at	Kcnj8	17.7
1428345_at	Ppapdc2	42.0	1436203_a_at	1110059G02Rik	16.4
1442698_at	---	41.1	1435751_at	Abcc9	16.3
1440926_at	Flt1	40.8	1424186_at	Ccdc80	15.4
1456061_at	Gimap8	40.7	1452474_a_at	Art3	15.2
1452013_at	Atp10a	40.6	1424902_at	Plxdc1	15.0
1418059_at	Eltd1	39.6	1424733_at	P2ry14	14.6
1438587_at	4933417M04Rik	39.3	1455050_at	E130203B14Rik	14.3
1441907_s_at	Cd93	38.9	1423477_at	Zic1	13.3
1440225_at	Gpr116	38.8	1448918_at	Slco3a1	13.1
1451500_at	Ushbp1	38.7	1448132_at	Slc19a1	12.8
1456152_at	Paqr5	38.6	1437165_a_at	Pcolce	12.8
1447584_s_at	Myct1	37.8	1448123_s_at	Tgfbi	12.5
1459888_at	LOC545261	37.6	1455271_at	LOC620695	12.5
1422028_a_at	Ets1	37.0	1417011_at	Sdc2	12.4
1457867_at	Sgpp2	36.9	1434893_at	Atp1a2	12.4
1417616_at	St6galnac2	36.8	1440879_at	Abca9	11.9
1418979_at	Akr1c14	36.5	1430700_a_at	Pla2g7	11.7
1420459_at	Dscr6	36.4	1424099_at	2310016C16Rik	11.7
1438027_at	5830443L24Rik	35.8	1422058_at	Nodal	11.7
1457141_at	Aqp11	35.7	1417534_at	Itgb5	11.6
1416832_at	Slc39a8	35.6	1419663_at	Ogn	10.1
1429177_x_at	Sox17	35.3	1422545_at	Tbx2	10.1
1456768_a_at	Mmrn2	35.0	1420872_at	Gucy1b3	10.0
1444181_at	Gimap5	35.0	1435741_at	Pde8b	9.9
1419758_at	Abcb1a	34.7	1454966_at	Itga8	9.8
1426914_at	Marveld2	34.5	1449860_at	Higd1b	9.4
1432176_a_at	Eng	34.3	1416271_at	Perp	8.8
1439221_s_at	Cd40	34.2	1420688_a_at	Sgce	8.7
1448862_at	Icam2	33.6	1430136_at	Grm3	8.2
1448471_a_at	Ctla2a	33.3	1435805_at	Lin7a	8.0
1435418_at	Slc22a8	33.0	1418157_at	Nr2f1	7.6

A) Most enriched CNS endothelial transcripts. List of the 50 most enriched endothelial cell transcripts defined as the greatest ratio of brain endothelial cell (GFP^+^ PDGFRbeta^−^): brain parenchyma (GFP^−^) values analyzed by affymetrix GeneChips.

B) Most enriched CNS pericyte transcripts. List of the 50 most enriched pericyte transcripts defined as the greatest ratio of brain vascular (GFP^+^): brain endothelial (GFP^+^ PDGFRbeta^−^) values analyzed by Affymetrix GeneChips.

### Identification of the Blood-Brain Barrier Transcriptome

**Figure 2:**
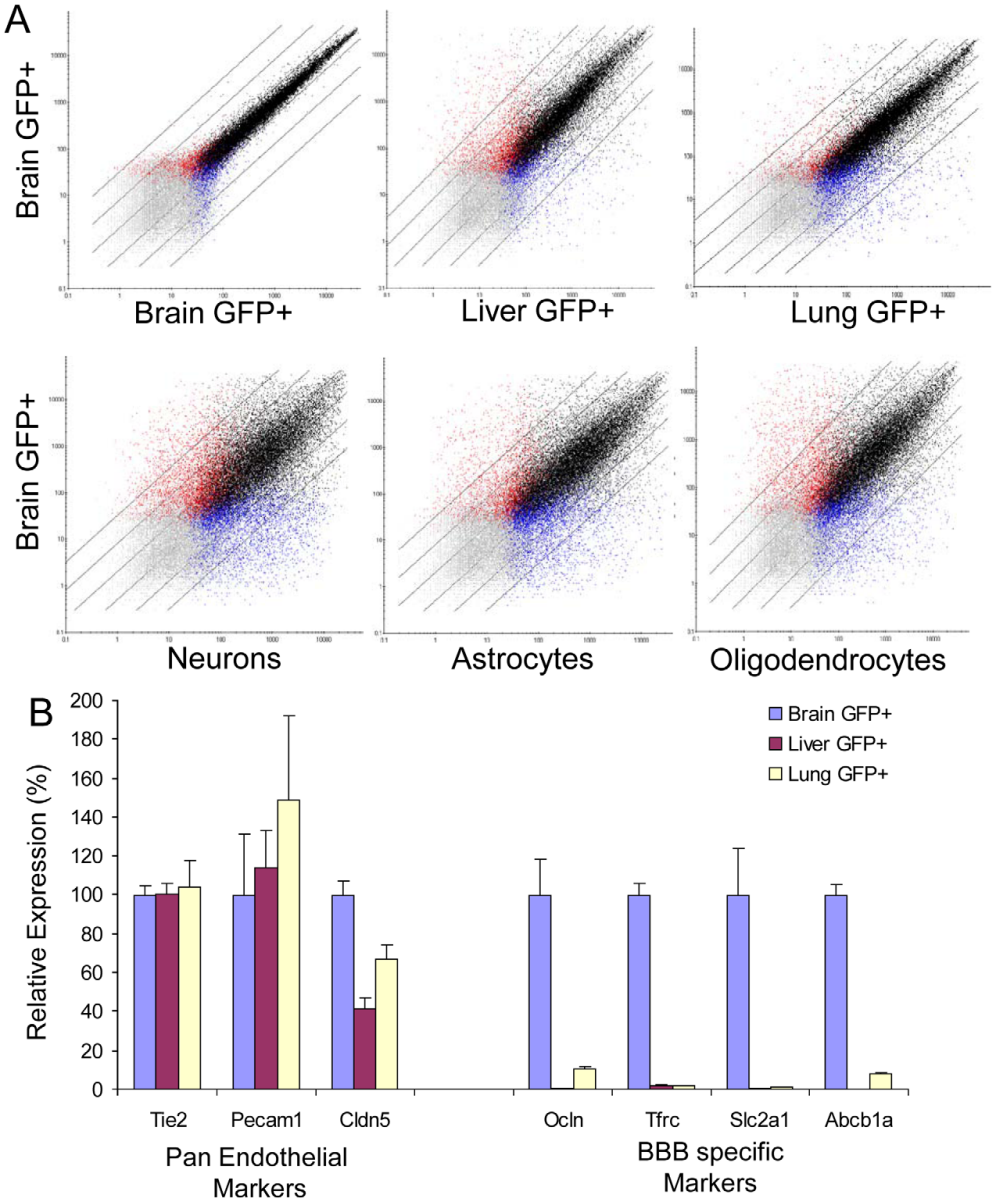
Comparison of transcriptional profiles of brain endothelial cells with liver and lung endothelial cells. A) Affymetrix GeneChips were utilized to compare gene expression in GFP^+^ cells isolated from cerebral cortex, liver and lung of Tie2GFP transgenic mice. The data is represented as a dot plot on a logarithmic scale, where each point reflects a probe set on the GeneChip. Black dots indicate probe sets identified as present in both samples, red dots indicate probe sets identified as present in the brain but not peripheral sample, blue dots indicate probe sets present in the peripheral but not brain sample, and grey dots represent probe sets identified as absent in both samples. Two biological replicates of the brain GFP^+^ samples were compared to identify variation between replicates. In addition, brain GFP^+^ cells were compared with profiles generated for neurons and glia by Cahoy et al (2008). Black diagonal lines represent cutoffs for two-fold, four-fold and eight-fold differences. B) Validation of transcriptional profiling by comparison of GeneChip expression with known CNS endothelial markers. Expression values are given relative to the brain GFP^+^ sample. Pan endothelial transcripts are expressed in all GFP^+^ samples, whereas BBB transcripts were enriched in the Brain GFP^+^ sample. Error bars represent SEM.

**Figure 3:**
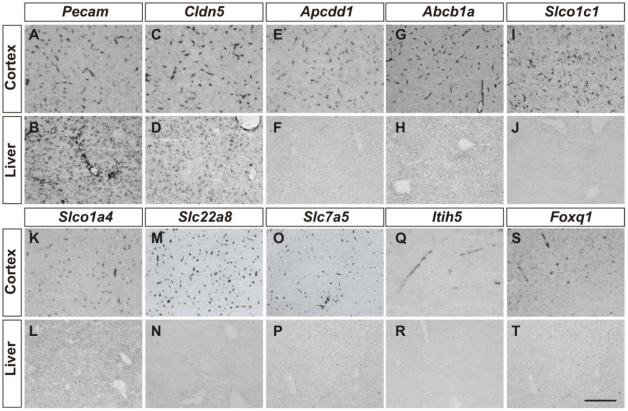
Selective expression of blood-brain barrier-enriched genes in CNS endothelial cells. Expression of *Pecam* (A, B), *Cldn5* (C, D), *Apcdd1* (E, F), *Abcb1a1* (G, H), *Slco1c1* (I, J), *Slco1a4* (K, L), *Slc22a8* (M, N), *Slc7a5* (O, P), *Itih5* (Q, R) and *Foxq1* (S, T) mRNA in the brain and liver of P20 mouse pups. The top four brain endothelial transcripts enriched compared with liver (Itih5, Apcdd1, Abcb1a and Slco1c1) and lung endothelial cells (Slc22a8, Slc7a5, Slco1a4 and Foxq1) were analyzed, excluding transcripts also expressed in the brain parenchyma (brain GFP^+^:brain GFP^−^<2), and those expressed at <10000 to ensure that the signal would be above the detection limit of the *in situ* hybridization. Note that *Pecam1 and Cldn5* mRNAs are expressed in the endothelial cells in both brain and liver. However, the transcripts for the blood-brain barrier enriched genes are only present in brain but not liver endothelial cells. Bar, 200 microns.

In order to identify blood-brain barrier enriched transcripts, we next utilized GeneChip analysis to compare the gene expression in GFP^+^ cells isolated from the brain, liver and lung of adult Tie2GFP mice. In each case, we used a double FACS purification procedure in order to isolate GFP^+^ cells for maximal purity. Comparison of the gene profiles of brain and liver, or brain and lung, revealed that the GFP^+^ cell populations in these tissues were very similar. This is graphically represented by a dot plot, in which each dot represents a probe set on the Affymetrix GeneChip, and their position relative to the logarithmic X and Y axes represents expression levels in the different tissues analyzed (Figure 2a). In these analyses, the vast majority of genes lie near the diagonal axis, indicating that their expression levels are not more than two-fold different between the samples compared. There are, however, outliers, genes which are expressed at vastly different levels in the vasculature of the different tissues. These differences were not identified between two biological replicates of brain GFP^+^ cells (Figure 2a). Furthermore, far greater variation was observed between the transcriptional profile of brain endothelial cells and those for neurons and glia (Figure 2a, data for neurons and glia generated by Cahoy et al 2008). The genes which are expressed at high levels in the brain vasculature, but low or absent from the liver and lung, are BBB-enriched genes. For BBB-enriched genes we identify genes that are enriched in the Brain GFP^+^ (vascular) cells, compared to liver GFP^+^ cells and lung GFP^+^ cells, and eliminate any pericyte enriched genes by comparing Brain GFP^+^PDGFRbeta^−^ endothelial cells with Brain GFP^+^ vascular cells. Preliminary validation of this data set comes from analyzing characterized endothelial genes. For instance, known pan-endothelial genes, including Tie2, PECAM1 and claudin 5, are expressed at similar levels in all tissue data sets, whereas established BBB-specific genes glut-1, p-glycoprotein, occludin and the transferrin receptor, are expressed at significantly higher levels in the brain vasculature than the liver or lung (Figure 2b). To further validate our data set we used *in situ* hybridization to analyze the expression pattern of the several top brain endothelial transcripts enriched compared with liver (Itih5, Apcdd1, Abcb1a and Slco1c1) and lung endothelial cells (Slc22a8, Slc7a5, Slco1a4 and Foxq1) (Figure 3). In each case, we observed expression in vascular cells in the brain but not liver. Interestingly, the pattern observed was different between the probe sets tested. For instance, Itih5 and Foxq1 appeared to be expressed sporadically and specifically in larger vessels, whereas Apcdd1, Slco1a4 and Slc22a8 appear to be expressed in smaller vessels. Indeed, hetereogeneity between the different segments of the CNS vasculature has been observed[16], and specific transcripts may be differentially expressed between arteries, arterioles, capillaries, venules and veins.

From this analysis, we conclude that we have generated a comprehensive data set representing the blood-brain barrier transcriptome. In Table 2 we list the most BBB enriched genes: those with the greatest difference between expression levels in the brain GFP^+^ cells versus liver and lung GFP^+^ cells, with parenchymal and pericyte/VSM transcripts excluded from these lists. All BBB enriched genes are listed in Table S6. In addition to identifying BBB enriched transcripts, we can also use this data set to identify genes which are enriched in the liver and lung endothelial cells compared with brain endothelial cells (Table S7). These genes might be important for regulating the permeability of non-CNS vessels, or imparting specific functions of non-CNS endothelial cells. In the following sections we present functional subsets of BBB enriched genes including tight junction proteins, transporters, signaling molecules and metabolic pathways that together constitute the blood-brain barrier.

**Table 2:** Most BBB enriched transcripts.

A.Probe Set ID	Gene Symbol	Brain/Liver	Brain/Lung	B.Probe Set ID	Gene Symbol	Brain/Liver	Brain/Lung
1441946_at	Itih5	3124.8	213.3	1418706_at	Slc38a3	85.5	821.1
1449070_x_at	Apcdd1	3087.4	75.2	1435418_at	Slc22a8	774.6	764.3
1419758_at	Abcb1a	1848.7	12.9	1418326_at	Slc7a5	15.8	734.2
1423343_at	Slco1c1	1745.2	310.2	1420405_at	Slco1a4	847.4	450.2
1437967_at	Titin	948.1	63.9	1423424_at	Zic3	438.5	441.2
1448147_at	Tnfrsf19	908.6	18.9	1438558_x_at	Foxq1	317.0	404.8
1420405_at	Slco1a4	847.4	450.2	1423343_at	Slco1c1	1745.2	310.2
1447787_x_at	Gja7	825.5	3.8	1428223_at	Mfsd2	777.8	224.9
1428223_at	Mfsd2	777.8	224.9	1441946_at	Itih5	3124.8	213.3
1435418_at	Slc22a8	774.6	764.3	1426215_at	Ddc	142.0	171.7
1418094_s_at	Car4	772.1	2.9	1434734_at	E130016E03Rik	168.2	167.1
1419700_a_at	Prom1	589.5	69.8	1453072_at	Gpr160	10.8	165.4
1429154_at	Slc35f2	552.5	69.2	1415802_at	Slc16a1	59.3	148.3
1447272_s_at	Atp10a	527.2	42.8	1442347_at	Lrp8	387.2	145.4
1429348_at	Sema3c	511.0	1.3	1449184_at	Pglyrp1	371.8	116.0
1448557_at	1200015N20Rik	481.7	10.9	1454734_at	Lef1	89.5	108.6
1428663_at	5133401H06Rik	472.0	4.8	1436417_at	Slc19a3	249.7	105.8
1425779_a_at	Tbx1	469.4	25.2	1426399_at	Vwa1	202.8	103.8
1423424_at	Zic3	438.5	441.2	1418383_at	Apcdd1	448.3	95.6
1457867_at	Sgpp2	429.8	9.3	1460459_at	Paqr5	72.3	80.2
1460465_at	A930038C07Rik	419.3	9.8	1439609_at	---	116.6	70.8
1423608_at	Itm2a	415.0	25.3	1419700_a_at	Prom1	589.5	69.8
1451255_at	MGI:1927471	412.6	41.0	1451047_at	Itm2a	266.5	69.5
1427157_at	E030025D05Rik	390.4	0.8	1429154_at	Slc35f2	552.5	69.2
1442347_at	Lrp8	387.2	145.4	1437967_at	Titin	948.1	63.9
1416301_a_at	Ebf1	380.1	11.8	1423858_a_at	Hmgcs2	63.9	62.7
1425415_a_at	Slc1a1	372.5	12.5	1417061_at	Slc40a1	1.1	59.2
1449184_at	Pglyrp1	371.8	116.0	1452661_at	Tfrc	48.1	55.4
1439505_at	Clic5	357.9	0.9	1435123_at	C030014M07Rik	52.6	52.2
1426431_at	Jag2	325.6	4.4	1424549_at	Degs2	156.4	50.0
1438558_x_at	Foxq1	317.0	404.8	1456140_at	Zic5	61.5	48.7
1448117_at	Kitl	261.9	1.2	1426599_a_at	Slc2a1	234.7	48.5
1448873_at	Ocln	255.5	9.8	1417714_x_at	Hba-a1	5.2	47.7
1438452_at	Nebl	253.1	4.7	1427931_s_at	Pdxk	31.0	43.7
1437360_at	Pcdh19	249.8	19.4	1447272_s_at	Atp10a	527.2	42.8
1436417_at	Slc19a3	249.7	105.8	1451255_at	Lsr	412.6	41.0
1454622_at	Slc38a5	237.6	13.5	1435026_at	Spock2	180.3	37.7
1426599_a_at	Slc2a1	234.7	48.5	1456147_at	st8sia6	95.7	36.9
1433933_s_at	Slco2b1	226.7	18.5	1426082_a_at	Slc16a4	94.0	34.0
1418412_at	Tpd52l1	216.4	25.6	1417022_at	Slc7a3	38.6	33.0
1454795_at	Cobll1	216.0	5.9	1435465_at	Kbtbd11	129.9	30.5
1426399_at	Vwa1	202.8	103.8	1456616_a_at	Bsg	24.7	30.1
1452968_at	Cthrc1	200.9	7.2	1452202_at	Pde2a	0.7	30.1
1459903_at	Sema7a	194.3	4.2	1417184_s_at	Hbb-b	3.0	29.0
1418979_at	9030611N15Rik	182.3	9.9	1442698_at	---	5.7	28.5
1435026_at	Spock2	180.3	37.7	1437268_at	Lancl3	53.2	28.0
1434734_at	Rad54b	168.2	167.1	1418412_at	Tpd52l1	216.4	25.6
1437479_x_at	Tbx3	163.6	1.8	1429206_at	Rhobtb1	2.7	25.4
1424549_at	Degs2	156.4	50.0	1451675_a_at	Alas2	6.1	25.4
1426215_at	Ddc	142.0	171.7	1451857_a_at	5730593N15Rik	31.2	25.3

The 50 most enriched transcripts in brain GFP^+^ cells compared with (A) liver, or (B) lung. Fold-enrichment given as a ratio of brain GFP^+^: peripheral GFP^+^ microarray expression values. To highlight BBB enriched transcripts, only probe sets ≥ two-fold enriched in brain endothelial cells compared with brain parenchyma (GFP^-^) are listed, and pericyte genes are excluded.

### Identification of Novel BBB Enriched Tight Junction Molecules

Tight junctions (TJs) are crucial components of the blood-brain barrier [17], but many of the key molecules that constitute these junctions are not yet known. In Table 3, we list the tight junction transcripts expressed by brain endothelial cells, and their relative expression levels in liver and lung endothelial cells. Among the claudin gene family, claudin 5 and claudin 12 are expressed by endothelial cells in all tissues, as described in the literature [18], [19]. Claudin 1, 3, and 11 have also been suggested to be expressed at the BBB [11], [20], [21], however, we did not see a definitive signal for any of these molecules. We also did not see expression of these molecules in CNS vessels by mRNA *in situ* hybridization and immunofluoresence imaging (data not shown), suggesting that claudin 5 and 12 are the key claudins expressed at the BBB. Whereas these endothelial claudins are expressed by endothelial cells in all tissues analyzed, we found that other tight junction transmembrane molecules including occludin, marveld2 (tricellulin), and Jam4 are highly enriched in brain endothelial cells. The specific expression of occludin at the BBB has been well documented [22], however, the enrichment of Jam4 and marveld2 in CNS endothelial cells is a novel finding. Marveld2 functions to seal tight junctions at points where three cells meet [23], [24]. The expression of this molecule by brain endothelial cells suggests that sealing tripartite adhesions may be critical for forming a high electrical resistance barrier. Endothelial cells of BBB capillaries form TJs with adjacent endothelial cells, as well as intra-cellular junctions as they fold over on to themselves forming the vessel tube. Thus, tripartite adhesions at the BBB could be the interface between intercellular tight junction strands and intracellular tight junction strands.

**Table 3:** BBB enriched tight junction molecules.

Probe set	Gene Symbol	Brain GFP+	Liver GFP+	Lung GFP+
**Transmembrane Molecules**				
1417839_at	Cldn5	38079.1	15781.7	25539.9
1433781_a_at	Cldn12	1728.6	1554.1	2732.0
1448873_at	Ocln	10303.7	40.3	1054.4
1451407_at	Jam4	1053.1	21.9	219.6
1436568_at	Jam2	11440.3	11372.0	3897.8
1426914_at	Marveld2	2877.8	72.7	554.4
**Cytoplasmic Adaptors**				
1450985_a_at	Tjp2	9089.6	3809.5	9019.6
1417749_a_at	Tjp1	25536.7	14771.9	16378.3
1426691_at	Tjap1	509.3	588.0	572.8
1455179_at	Mpp7	787.1	195.1	436.3
1434954_at	Mpp5	1197.4	1166.8	884.7
1450919_at	Mpp1	1438.7	3789.9	1506.7
1435461_at	Magi3	3487.4	1785.9	2131.1
1452309_at	Cgnl1	8039.7	430.1	1562.4
1415691_at	Dlg1	3574.6	2188.6	3425.2
1426465_at	Dlgap4	773.3	1219.4	1842.6
1427064_a_at	Scrib	551.2	605.5	625.0
1420851_at	Pard6g	1176.7	426.6	4731.0
1434775_at	Pard3	2325.2	62.8	1465.7
1450937_at	Lin7c	2913.0	2055.6	2396.9
1424595_at	F11r	4332.0	5220.9	4775.5
1460356_at	Esam1	21470.6	16937.8	15387.4
1433956_at	Cdh5	2893.8	4790.3	6622.2
1420985_at	Ash1l	2011.1	1239.5	2094.6
1426623_a_at	Arhgap17	845.4	1530.9	891.0
1452387_a_at	Amotl2	2714.8	5359.0	2375.6
1428785_at	Amotl1	1989.5	6058.1	2486.1
1454890_at	Amot	5943.5	3708.8	835.5
1433676_at	Wnk1	15543.9	10052.6	12021.5

Transcripts defined by MGI as tight junction molecules, or related family members, expressed in brain GFP^+^ cells with an expression value ≥500 by Affymetrix GeneChip analysis. Transcripts highlighted in red are enriched at least 3-fold over liver endothelial cells.

Several intracellular accessory tight junction molecules are also expressed in all endothelial cells. These include ZO-1, ZO-2, Mpdz, MPP1, MPP5, MPP6, MPP7, Dlgh1, Dlgh5, Dlgap4, lglh and Scrib. These proteins may play integral roles in linking the tight junction to the actin cytoskeleton as well as regulating the function of the junctions. However, they are expressed by all endothelial cells, and therefore their presence alone does not define the BBB. In our data set we have also identified several interesting cytoplasmic tight junction molecules as being highly enriched at the BBB, including cingulin-like 1 (jacop) and pard3. Cingulin-like 1 is also of great interest as it is homologous to cingulin, which links tight junctions to the actin cytoskeleton [25]. Perhaps the difference between peripheral and CNS endothelial cells is not the expression of the structural claudins, but in how claudins are regulated by the cytoskeleton. The pard family molecules are highly conserved throughout evolution from worms to flies to mammals and are involved in the formation of asymmetry in cells during development [26], [27]. This is very interesting as a defining feature of blood-brain barrier forming endothelial cells is that the tight junctions form highly asymmetric cells, and that different transporters are localized specifically to the luminal or abluminal surface [28].

In each case, the BBB enriched TJ molecules identified, whether occludin, marveld2, jam4, cingulin-like 1, or pard3, all have far greater expression levels in the brain endothelial cells than liver endothelial cells, with lung endothelial cells expressing at intermediate levels. This pattern follows the known level of permeability of the vessels in each tissue. BBB vessels form a tight impermeable barrier, liver sinusoidal capillaries are among the leakiest vessels in the body, whereas lung vessels have an intermediate permeability [29], [30]. The fact that the expression levels of enriched tight junction molecules follows this pattern suggests that we may be able to identify novel molecules involved in barrier function by examining our data set for probe sets whose expression level increases in the liver GFP^+^ to lung GFP^+^ to brain GFP^+^ microarray analyses. These quantitative profiles should be highly useful for identifying candidate genes that may regulate the differential permeability observed in blood vessels of various tissues throughout the body. With this in mind, novel molecules that follow a similar pattern may also be important for BBB formation. A number of these molecules are known to associate with cellular junctions in other tissues including Sorbs1, Sorbs2, Enah, Neo1 and Lims2, and thus may be important for BBB formation [31], [32], [33], [34], [35]. In addition we may be able to identify novel transmembrane molecules involved in structural adhesions. Intriguing candidates are Itm2a, Apcdd1, Tmeff1, Tmem23, Ly75, Ly6c, cd34, Pcdh19 and Extl3. Also, several Golgi protein glycosylation enzymes, including St6galnac2, St8sia4 and st8sia6, are identified using this analysis, suggesting that processing of proteins may be important in regulating BBB formation.

### Elucidation of the Set of BBB-Enriched Transporters

Another key aspect of the blood-brain barrier is the expression of transporters by CNS endothelial cells. Whereas many transporters have already been identified as highly enriched at the BBB, a genome wide analysis has not been performed before. In Table 4 we present a list of transporters expressed by the CNS vasculature. We divide these into four categories: BBB enriched transporters which are enriched in CNS endothelial cells compared with peripheral endothelial cells; pan-endothelial transporters identified in all endothelial cells analyzed; pericyte enriched transporters (Table 4); and ubiquitous transporters expressed by all cells analyzed (data not shown). Elucidating the substrates of these transporters may provide a greater understanding of how endothelial cells participate in the metabolic division of labor within the CNS. Furthermore, these transporters may be utilized to target specific molecules for delivery into the brain for therapeutic purposes.

**Table 4:** Transporters of the BBB.

A.Probe set ID	Gene Symbol	Brain GFP+	Liver GFP+	Lung GFP+	B.Probe set ID	Gene Symbol	Brain GFP+	Liver GFP+	Lung GFP+
1433933_s_at	Slco2b1	7798.5	34.4	420.7	1439368_a_at	Slc9a3r2	11220.0	5884.7	9141.2
1423343_at	Slco1c1	30482.2	17.5	98.3	1438559_x_at	Slc44a2	7412.4	30206.2	17458.8
1420405_at	Slco1a4	38443.9	45.4	85.4	1433645_at	Slc44a1	8029.7	5969.1	5828.9
1418326_at	Slc7a5	13681.2	866.3	18.6	1425364_a_at	Slc3a2	18715.9	8790.5	12701.4
1417022_at	Slc7a3	2336.3	60.5	70.8	1416832_at	Slc39a8	11166.0	16155.8	1380.6
1454991_at	Slc7a1	31330.8	696.7	2873.3	1429593_at	Slc38a2	3477.9	2701.2	3444.1
1437149_at	Slc6a6	21228.3	1608.7	6912.5	1452059_at	Slc35f5	1790.0	1341.4	2176.9
1436137_at	Slc6a17	243.6	114.6	98.1	1453300_at	Slc35d2	1199.2	912.8	421.2
1451486_at	Slc46a3	2002.6	343.5	392.6	1433898_at	Slc25a30	1114.3	1358.5	556.7
1447227_at	Slc40a1	953.7	116.1	22.8	1424735_at	Slc25a25	677.4	578.3	766.9
1433751_at	Slc39a10	36219.1	1987.1	3932.0	1452717_at	Slc25a24	3275.4	2221.4	2520.1
1454622_at	Slc38a5	5337.6	22.5	396.4	1450395_at	Slc22a5	2034.3	1478.3	1606.7
1418706_at	Slc38a3	8758.2	102.4	10.7	1429726_at	Slc16a9	6134.7	164.5	4913.5
1429154_at	Slc35f2	3223.2	5.8	46.6	1418257_at	Slc12a7	1271.8	1519.8	2671.3
1433750_at	Slc31a1	3655.1	1091.9	953.6	1451227_a_at	Slc10a3	1095.1	1157.3	620.5
1436164_at	Slc30a1	23687.8	3827.4	4884.1	1418774_a_at	Atp7a	2948.3	2002.4	3669.4
1434773_a_at	Slc2a1	41376.6	267.8	1419.6	1442145_at	Atp13a3	534.7	250.3	2057.8
1424211_at	Slc25a33	936.5	332.8	188.8	1455083_at	Atp11c	4660.2	3234.7	3048.5
1453149_at	Slc25a32	1131.7	368.3	253.8	1451388_a_at	Atp11b	5863.6	1539.2	2495.8
1423109_s_at	Slc25a20	1082.6	408.0	515.9	1436544_at	Atp10d	3373.8	1319.2	2658.9
1435418_at	Slc22a8	9605.2	12.4	12.6					
1448299_at	Slc1a1	8321.1	61.9	521.2					
1436417_at	Slc19a3	9686.6	38.8	91.6					
1426082_a_at	Slc16a4	1724.0	18.3	50.8					
1418445_at	Slc16a2	3708.5	689.0	232.1					
1415802_at	Slc16a1	32259.1	544.3	217.5					
1447851_x_at	Atp10a	16691.2	52.4	676.0					
1422906_at	Abcg2	24521.4	2038.1	4007.5					
1443870_at	Abcc4	17102.3	2475.8	872.6					
1419759_at	Abcb1a	42011.6	129.7	3931.4					

C.Probe Set ID	Gene Symbol	Brain Vascular	Brain Endothelial	Vascular/Endothelial					
1448918_at	Slco3a1	474.1	36.3	13.1					
1438751_at	Slc30a10	824.0	42.1	19.6					
1448132_at	Slc19a1	1108.0	86.4	12.8					
1417622_at	Slc12a2	8112.9	2419.8	3.4					
1421129_a_at	Atp2a3	1456.1	652.0	2.2					
1435148_at	Atp1b2	1477.6	247.5	6.0					
1434893_at	Atp1a2	2744.3	221.9	12.4					
1436411_at	Atp13a5	5209.0	260.3	20.0					
1435751_at	Abcc9	7314.9	448.6	16.3					
1440879_at	Abca9	630.6	53.0	11.9					

A) BBB enriched transporters. Transporters from the Slc, Slco, ATP and Abc gene families with enriched expression in brain endothelial cells. Transporters identified in Table S6, all of which have at least a two-fold greater expression value in brain GFP^+^ than liver and lung GFP^+^ cells, pericyte genes excluded.

B) Endothelial transporters. Transporters expressed in multiple endothelial samples. These transporters were identified as endothelial specific in Table S2, but were not significantly enriched in brain GFP^+^ cells compared to either the liver GFP^+^ or lung GFP^+^ samples.

C) Pericyte enriched transporters. A list of pericyte enriched transporters from Table S3, defined as those transcripts whose GeneChip expression values have a brain vascular:endothelial ratio of ≥2.

### Identification of Highly BBB-Enriched Signaling and Metabolic Pathways

The molecular signals that regulate the formation and function of the BBB remain largely unknown. We therefore took advantage of Ingenuity Pathway Analysis (IPA) software to analyze the CNS endothelial enriched transcripts to identify signaling pathways that are enriched at the BBB or in peripheral endothelial cells (Table 5a,c). We identified several pathways as being significantly associated with brain endothelial cells, including the Wnt/beta-catenin signaling pathway[36]. This pathway has been demonstrated to be specifically activated in CNS endothelial cells, and is important for regulating CNS-specific angiogenesis and inducing BBB properties including the expression of glut-1 and tight junction proteins[36], [37], [38]. The identification of the BBB inducing Wnt pathway validates this database as a method to identify pathways that may be important for BBB formation.

**Table 5:** Signaling cascades and metabolic pathways enriched at the BBB and peripheral endothelial cells.

A.BBB enriched signaling pathway	P-value	B.BBB enriched metabolic pathway	P-value
LPS/IL-1 Mediated Inhibition of RXR Function	1.20E-05	Glycosphingolipid Biosynthesis - Neolactoseries	0.004
Serotonin Receptor Signaling	0.001	Glycine, Serine and Threonine Metabolism	0.008
Clathrin-mediated Endocytosis Signaling	0.003	Phenylalanine Metabolism	0.010
Dopamine Receptor Signaling	0.008	Nitrogen Metabolism	0.025
PXR/RXR Activation	0.025	Glycolysis/Gluconeogenesis	0.027
Regulation of IL-2 Expression in Activated and Anergic T Lymphocytes	0.039	Synthesis and Degradation of Ketone Bodies	0.031
Role of Wnt/GSK-3beta Signaling in the Pathogenesis of Influenza	0.039	Histidine Metabolism	0.035
Wnt/beta-catenin Signaling	0.040	Methionine Metabolism	0.035
Type II Diabetes Mellitus Signaling	0.049		

C.Peripheral enriched signaling pathway	P-value	D.Peripheral enriched metabolic athway	P-value
Hepatic Fibrosis / Hepatic Stellate Cell Activation	1.60E-07	Sphingolipid Metabolism	0.044
Dendritic Cell Maturation	3.40E-06		
Type I Diabetes Mellitus Signaling	1.20E-05		
Atherosclerosis Signaling	1.30E-04		
Hepatic Cholestasis	3.50E-04		
Complement System	3.70E-04		
Molecular Mechanisms of Cancer	6.80E-04		
Acute Phase Response Signaling	0.001		
Caveolar-mediated Endocytosis Signaling	0.001		
Role of Osteoblasts, Osteoclasts and Chondrocytes in Arthritis	0.001		
Death Receptor Signaling	0.001		
Production of NO and Reactive Oxygen Species in Macrophages	0.002		
IL-10 Signaling	0.002		
TNFR1 Signaling	0.002		
Macropinocytosis Signaling	0.002		
Leukocyte Extravasation Signaling	0.003		
Apoptosis Signaling	0.003		
Role of Macrophages, Fibroblasts and Endothelial Cells in Arthritis	0.003		
IL-8 Signaling	0.003		
NF-κB Signaling	0.003		
Antigen Presentation Pathway	0.003		
Integrin Signaling	0.004		
LPS/IL-1 Mediated Inhibition of RXR Function	0.004		
p53 Signaling	0.004		
Glioma Invasiveness Signaling	0.004		
HER-2 Signaling in Breast Cancer	0.006		
VDR/RXR Activation	0.007		
Colorectal Cancer Metastasis Signaling	0.008		
Graft-versus-Host Disease Signaling	0.009		
Altered T Cell and B Cell Signaling in Rheumatoid Arthritis	0.011		
IL-9 Signaling	0.011		
Virus Entry via Endocytic Pathways	0.012		
TWEAK Signaling	0.014		
LXR/RXR Activation	0.014		
Aryl Hydrocarbon Receptor Signaling	0.015		
HMGB1 Signaling	0.017		
Airway Pathology in Chronic Obstructive Pulmonary Disease	0.021		
Endothelin-1 Signaling	0.023		
Docosahexaenoic Acid (DHA) Signaling	0.023		
Cholecystokinin/Gastrin-mediated Signaling	0.024		
Induction of Apoptosis by HIV1	0.027		
Role of PKR in Interferon Induction and Antiviral Response	0.028		
Germ Cell-Sertoli Cell Junction Signaling	0.029		
CXCR4 Signaling	0.031		
Sphingosine-1-phosphate Signaling	0.032		
Retinoic acid Mediated Apoptosis Signaling	0.033		
GM-CSF Signaling	0.033		
IL-12 Signaling and Production in Macrophages	0.040		
Allograft Rejection Signaling	0.040		
TNFR2 Signaling	0.046		
Autoimmune Thyroid Disease Signaling	0.046		
T Helper Cell Differentiation	0.049		
Toll-like Receptor Signaling	0.049		

A–B) BBB enriched pathways. Ingenuity Pathway Analysis (IPA) software was utilized to identify signaling cascades (A) and metabolic pathways (B) that are significantly enriched at the BBB. For this analysis BBB enriched transcripts identified in Table S6 were utilized. Pathways in which p-value <0.05 are displayed.

C–D) Peripheral vascular enriched pathways. IPA software was utilized to identify signaling cascades (C) and metabolic pathways (D) that are significantly enriched in peripheral vessels compared to brain vessels. For this analysis peripheral enriched transcripts identified in Table S7 were utilized. Pathways in which p-value <0.05 are displayed.

The “LPS/IL-1 mediated inhibition of retinoic x receptor (RXR) function” pathway was enriched with the highest confidence level in the CNS endothelial cells compared with liver and lung endothelial cells. The core of this pathway is a transcriptional cassette activated by the nuclear receptor RXRalpha, which can also be inhibited by a cytoplasmic kinase cascade initiated by LPS, IL-1 or TNFalpha signals. Many transcripts whose expression is turned on by RXRalpha are specifically expressed by CNS endothelial cells. These transcripts include Oatp2, Mrp4, Mdr1, Aldh, Pltp, Cpt, Hmgcs and Alas1, some of which are BBB enriched transporters presented in Table 4. Remarkably, the “LPS/IL-1 mediated inhibition of RXR function” pathway is also enriched in peripheral endothelial cells compared with brain endothelial cells. However, the transcripts enriched in peripheral endothelial cells encode molecules which inhibit RXRalpha function, including secreted molecules, membrane receptors and cytoplasmic signaling components (Lbp, IL-1, Cd14, Myd6b, Nfp). This suggests that a fundamental difference between CNS and peripheral endothelial cells is that RXRalpha mediated transcription is activated in CNS endothelial cells and inhibited in peripheral endothelial cells. In addition we have identified that neurotransmitter signaling, regulation of IL-2, and other pathways are enriched in brain endothelial cells. These findings strongly suggest in addition to Wnt signaling, RXRalpha, and other signaling may impart BBB specific properties to CNS endothelial cells.

IPA software was also used to identify metabolic pathways enriched at the BBB or in peripheral endothelial cells (Table 5b,d). Glycolysis/gluconeogenesis and amino acid metabolism are enriched at the BBB, suggesting that brain endothelial cells may be intimately involved in the production of energy metabolites and amino acids for neurons, a function that has been thought to be uniquely served by astrocytes.

### Prediction of Novel Cell-Cell Signaling Interactions Between Vascular and Parenchymal Cell Types

From the endothelial and pericyte transcriptome data sets, we next generated predictions about the molecular nature of the cell-cell interactions by which endothelial cells and pericytes communicate. These interactions may be critical for vascular contraction, remodeling, differentiation, and permeability. In fact of the top 50 pericyte enriched genes, 40 are predicted by David Bionformatics or Mouse Genome Informatics (MGI) to encode transmembrane or secreted proteins, and thus are potentially important for endothelial-pericyte signaling. Using this analysis we identified previously known endothelial-pericyte signals, thus validating this approach. For instance, endothelial secreted PDGF-BB regulates the proliferation and survival of CNS pericytes through PDGFRbeta as evidenced by the complete lack of CNS pericytes in PDGFB or PDGFRbeta deficient mice [39], [40]. In our data set, PDGFB is enriched in endothelial cells whereas PDGFRbeta is specifically expressed by pericytes. Other potential endothelial-to-pericyte signals include endothelial-derived Jagged2 or Dll4 activation of pericyte-expressed Notch3, or endothelial-derived sema3c, sema3g, sema4c and sema7a signaling to pericyte-expressed plexin B2, Plxdc1 and Plxdc2. Putative pericyte-to-endothelial signals include pericyte derived TGFbeta superfamily members bmp5 and nodal, and endothelial cell expressed activin receptors (Acvr1, Acvrl1) and bmp receptors (Bmpr2). In addition we identified angiotensin I converting enzyme 2 (Ace2) as highly expressed by pericytes, and thus likely to be involved in generating ligands for agtrl1 receptors made by endothelial cells. These ligand-receptor pairs are thus candidates to play important roles in brain angiogenesis and BBB development.

We further looked for novel vascular-parenchymal cell interactions. Recent work suggests that neural-vascular cellular interactions are important for the development of the nervous system. Comparing the vascular enriched genes with those expressed by neurons and glia [14] can lead to the discovery of signaling pathways important for the development of the CNS. Astrocytes possess long processes whose endfeet ensheath blood vessels. This close association has been implicated in regulating angiogenesis, vascular contraction and blood-brain barrier permeability [41]. Among astrocyte enriched genes defined by Cahoy et al (2008), 798 of 2618 are predicted by DAVID bioinformatics to be membrane or secreted. Of these, many have putative receptors expressed by endothelial cells. For instance putative astrocytes/endothelial interactions may be mediated through ApoE/Lrp8; BMP7/ActrIIa; AGT/Agtrl1; Wnt7a,Wnt7b/Fzd4,Fzd6; ang1/tie-2, and VEGFa/Flt1, Flt, Kdr. Blood vessels also interact with neural stem cells in the developing and adult nervous system, providing a “vascular niche” that maintains stem cells, promotes their division, and expands neurogenesis [42]. Along these lines, we identified a high level of expression of potential oligodendrocyte precursor cell (OPC) mitogens within endothelial cells including cxcl12/sdf1, kitl, csf1, and PDGFB, with cognate receptors being highly expressed by OPCs, respectively cmkor1/cxcr7, c-kit, csf1r, and PDGFRalpha [43], [44], [45], [46].

### Developmental Changes in BBB Gene Expression

In order to understand developmental changes that regulate angiogenesis and formation of the BBB, we used FACS to purify and then profile vascular cells (GFP^+^) from the cerebral cortex of Tie2GFP mice of different ages. We compared cells purified from postnatal (P2–8) mice and adult (p60–70) mice. In Table S4 and Table S5 we list the CNS vascular transcripts significantly up-regulated and down-regulated during development from postnatal to adult mice, and in Table 6 we list the signaling cascades and metabolic pathways identified by IPA that significantly change during development. Interestingly, Interferon, Prolactin, Growth Hormone, and circadian rhythm signaling are significantly up-regulated during development suggesting these pathways may regulate the maturation of the vasculature. On the other hand, integrin signaling, protein ubiquitination and hypoxia signaling are all down-regulated during development suggesting that these pathways may be involved in regulating angiogenesis.

**Table 6:** Developmentally regulated brain endothelial signaling cascades and metabolic pathways.

A.Up-regulated Signaling Pathway	P-value	C.Down-regulated Signaling Pathway	P-value
Interferon Signaling	1.60E-04	Mitochondrial Dysfunction	4.50E-05
Prolactin Signaling	3.90E-04	Airway Pathology in Chronic Obstructive Pulmonary Disease	0.004
Growth Hormone Signaling	0.001	RAN Signaling	0.004
Circadian Rhythm Signaling	0.001	Parkinson's Signaling	0.006
Synaptic Long Term Potentiation	0.003	Sphingosine-1-phosphate Signaling	0.006
Phospholipase C Signaling	0.005	Molecular Mechanisms of Cancer	0.007
IL-3 Signaling	0.007	Endothelin-1 Signaling	0.007
Cholecystokinin/Gastrin-mediated Signaling	0.007	Integrin Signaling	0.010
Factors Promoting Cardiogenesis in Vertebrates	0.008	Apoptosis Signaling	0.011
Renin-Angiotensin Signaling	0.009	Regulation of eIF4 and p70S6K Signaling	0.017
Molecular Mechanisms of Cancer	0.009	p70S6K Signaling	0.017
NRF2-mediated Oxidative Stress Response	0.010	Cell Cycle Regulation by BTG Family Proteins	0.018
GNRH Signaling	0.011	mTOR Signaling	0.018
JAK/Stat Signaling	0.011	Protein Ubiquitination Pathway	0.019
IGF-1 Signaling	0.012	Cytotoxic T Lymphocyte-mediated Apoptosis of Target Cells	0.029
Thrombopoietin Signaling	0.017	Glioma Invasiveness Signaling	0.030
Huntington's Disease Signaling	0.017	Hypoxia Signaling in the Cardiovascular System	0.032
Hypoxia Signaling in the Cardiovascular System	0.021	IL-8 Signaling	0.032
Role of Macrophages, Fibroblasts and Endothelial Cells in Arthritis	0.025	Production of NO and Reactive Oxygen Species in Macrophages	0.035
FLT3 Signaling in Hematopoietic Progenitor Cells	0.026	Death Receptor Signaling	0.042
BMP signaling pathway	0.026	Induction of Apoptosis by HIV1	0.045
EGF Signaling	0.029	Fcγ Receptor-mediated Phagocytosis in Macrophages	0.049
p53 Signaling	0.030	HMGB1 Signaling	0.049
Glioblastoma Multiforme Signaling	0.034		
Cardiomyocyte Differentiation via BMP Receptors	0.035		
Endoplasmic Reticulum Stress Pathway	0.035		
VDR/RXR Activation	0.036		
Corticotropin Releasing Hormone Signaling	0.036		
Retinoic acid Mediated Apoptosis Signaling	0.039		
Human Embryonic Stem Cell Pluripotency	0.041		
p38 MAPK Signaling	0.041		
Prostate Cancer Signaling	0.044		
Ephrin Receptor Signaling	0.046		
Melanocyte Development and Pigmentation Signaling	0.046		
Aryl Hydrocarbon Receptor Signaling	0.046		
Oncostatin M Signaling	0.048		
CNTF Signaling	0.048		
Activation of IRF by Cytosolic Pattern Recognition Receptors	0.048		
FGF Signaling	0.049		

B.Up-regulated Metabolic Pathway	P-value	D.Down-regulated Metabolic Pathway	P-value
Riboflavin Metabolism	0.022	Oxidative Phosphorylation	0.001
		Pentose Phosphate Pathway	0.001
		Methane Metabolism	0.004
		Phenylalanine Metabolism	0.004
		Purine Metabolism	0.010
		Lysine Degradation	0.020
		Fatty Acid Elongation in Mitochondria	0.036
		N-Glycan Degradation	0.046

A–D) IPA software analysis of developmentally regulated (2-fold up or down) transcripts, identifies signaling cascades (A,C) and metabolic pathways (B,D) that are up-regulated (A,B) or down regulated (C,D) during development. For this analysis developmentally regulated pathways identified in Table S4 (up-regulated) and Table S5 (down-regulated) were utilized. Pathways with p-value<0.05 are listed.

## Discussion

### Identification of the Blood-Brain Barrier Transcriptome

In this paper we provide a new resource for understanding gene expression in the developing and adult CNS vasculature. Previous gene profiling studies of brain vessels had several limitations [9], [10], [11], [47] including use of whole vessel fractions, which contain many different cell types, and the lack of elucidation of peripheral endothelial profiles for comparison. We have combined FACS purification with GeneChip analysis to generate a transcriptional profile of highly purified endothelial cells from the CNS, and through comparison with liver and lung endothelial cells we have generated a comprehensive list of transcripts enriched at the BBB. These transcripts encode tight junction molecules, transporters, metabolic enzymes, signaling cascades, and proteins of unknown function, that together form the highly specialized interface between the blood and the brain. This represents a novel resource for three reasons. First, we separate CNS endothelial cells from pericytes/VSM, and thus generate a transcriptional profile of endothelial cells, the key cells that form the BBB. Second, we compare brain endothelial cells with peripheral endothelial cells isolated from the liver and lung, identifying transcripts which are specific to barrier forming brain endothelial cells. Finally, we have isolated cells from different ages and thus can identify developmental changes in the BBB. The BBB transcriptome database should be highly useful for understanding the molecular control of CNS angiogenesis, BBB permeability, and the cell-cell interactions in the developing CNS.

### Identification of Candidates Transcripts that Control Vascular Permeability

A hallmark of the BBB is that CNS endothelial cells are held together by high electrical resistance tight junctions [17], [48]. Of the best characterized tight junction molecules expressed at the BBB, claudin 5, occludin, ZO-1 and ZO-2, only occludin is specifically expressed at the blood-brain barrier and not by non-barrier forming vessels [18], [22]. The fact that occludin deficient mice still form a functional BBB suggests that the expression of this molecule does not impart BBB function [49]. It therefore remains a mystery as to what causes CNS endothelial cells to form these high electrical resistance tight junctions. In this study we have identified several tight junction molecules whose expression is enriched at the BBB, including marveld2, cingulin-like-1, and pard3, that may play a crucial role in the formation of BBB tight junctions. Our data sets will also be useful to identify novel molecules not previously known to be associated with tight junction proteins, which may be important for BBB integrity. In addition to the highly specialized vasculature of the blood-brain barrier, there is great heterogeneity in the structure and function of vessels in all tissues [29], [30]. By analyzing the relative levels of transcripts expressed by the endothelial cells in different tissues we can generate hypotheses as to the relevance of different genes for the tissue specific functions of each vessel, including permeability, leukocyte trafficking, hemostasis, angiogenesis, immunity, and vasomotor tone.

### Signaling Pathways Enriched at the BBB

Transplantation experiments have established that the brain environment signals brain endothelial cells to form the BBB [5], but the identity of these signals are largely unknown. In particular, we have identified transcripts from a number of cascades that are enriched in the vasculature of the CNS including Wnt/beta-catenin, RXRalpha and other signaling pathways. The identification of the Wnt pathway, a known BBB inducing signaling pathway, validates this database as a method to identify pathways that may be important for BBB formation.

RXRalpha is a nuclear receptor which activates the transcription of a series of molecules. The activity of RXRalpha can be inhibited by a cytoplasmic kinase cascade initiated by LPS, IL-1 or TNFalpha signals [50]. Remarkably, genes in the inhibitory cassette of this pathway, including Lbp, IL-1, Cd14, Myd6b, Nfp are enriched in peripheral endothelial cells, whereas transcripts downstream of RXRalpha are enriched in CNS endothelial cells. Therefore, a fundamental difference between CNS and peripheral endothelial cells is the expression of the RXRalpha transcriptional cassette in CNS endothelial cells, and its suppression in peripheral endothelial cells. RXRalpha is a retinoic acid nuclear receptor which raises the question: Does retinoic acid (RA) regulate the BBB? This seems possible as addition of RA to endothelial cell lines *in vitro* increases the expression of BBB specific molecules Pgp and gamma-glutamyl transpeptidase [51], [52]. This matches our result that many of the RXRalpha regulated transcripts are the BBB enriched transporters, including Oatp2, Mdr1 and Mrp4. Interestingly, this pathway is inhibited by immune system modulating cytokines (IL-1) and bacterial LPS. Perhaps infection and inflammation alter the levels of BBB specific molecules through regulation of RXRalpha activity.

### Targeting Tumor Angiogenesis

In addition to identifying novel barrier forming molecules, our data also identifies transcripts that may be important for angiogenesis and/or vascular pathfinding. This is evident as we have identified integrins and semaphorins, both of which have been implicated in the formation of new vessels [53], [54], [55]. Identifying such molecules may be important for developing treatments for brain tumors, as formation of new vessels is a critical step in tumor progression. Disruption of tumor angiogenesis, without harming mature vessels, is a powerful technique for limiting tumor growth [56], [57]. To accomplish this, it is key to identify potential targets found on angiogenic vessels, that are absent from mature vessels or parenchymal tissue. In this resource we have identified transcripts whose expression level changes from developmental stages where robust angiogenesis is occurring, to adult animals where angiogenesis is largely completed. Potential targets include prion protein doublet (prnd), madcam1, tachykinin receptor 3, Slc7a8, and Cxcr4. Each of these transcripts is enriched in postnatal brain endothelial cells as compared with adult brain, liver and lung endothelial cells, and adult CNS parenchyma.

In summary, we have generated a comprehensive data set describing the transcriptome of the BBB. This will provide a valuable resource for understanding the development and function of this crucial barrier, as well as its role modulating CNS function.

## Supporting Information

Table S1 Complete Excel spreadsheet. The Excel spreadsheet containing the comprehensive list of probe sets from the mouse 430-2 GeneChip, with expression values for adult brain GFP^−^, adult brain GFP^+^, adult brain GFP^+^PDGFRbeta^−^, liver GFP^+^, lung GFP^+^, and postnatal brain GFP^+^ samples. Each expression value is the average of at least three biological replicates.
(9.96 MB XLS)

Table S2 Endothelial specific genes. A data set containing probe sets from the mouse 430–2 GeneChip statistically enriched in CNS endothelial cells compared with CNS parenchyma as identified by probe sets with GFP^+^PDGFRbeta^−^:GFP^−^ >2 with expression value in GFP^+^PDGFRbeta^−^>200. SAM algorithms were utilized to identify significance.
(0.87 MB XLS)

Table S3 Pericyte specific genes. A data set containing probe sets from the mouse 430–2 GeneChip statistically enriched in CNS pericytes compared with CNS endothelial cells as identified by probe sets with GFP^+^:GFP^+^PDGFRbeta^−^>2 with expression value in GFP^+^>200. SAM algorithms were utilized to identify significance.
(0.10 MB XLS)

Table S4 Developmentally up-regulated CNS vascular genes. A data set containing probe sets from the mouse 430–2 GeneChip statistically up-regulated in CNS vessels during development as identified by probe sets with adult GFP^+^:postnatal>2 with expression value in adult GFP^+^>200. Pericyte genes were eliminated from this dataset. SAM algorithms were utilized to identify significance.
(0.43 MB XLS)

Table S5 Developmentally down-regulated CNS vascular genes. A data set containing probe sets from the mouse 430–2 GeneChip statistically down-regulated in CNS vessels during development as identified by probe sets with adult GFP^+^:postnatal<2 with expression value in postnatal GFP^+^>200. Pericyte genes were eliminated from this dataset. SAM algorithms were utilized to identify significance.
(0.40 MB XLS)

Table S6 BBB enriched genes. A data set containing probe sets from the mouse 430–2 GeneChip statistically enriched in CNS endothelial cells compared with those from the CNS parenchyma as identified by probe sets with BrainGFP^+^:LiverGFP^+^>2 and BrainGFP^+^:LungGFP^+^>2 with expression value in BrainGFP^+^>200. Additionally separate work sheets are presented for data sets containing BrainGFP^+^:LiverGFP^+^>2 with expression value in BrainGFP^+^>200, and BrainGFP^+^:LungGFP^+^>2 with expression value in BrainGFP^+^>200. Pericyte genes were eliminated from each dataset. SAM algorithms were utilized to identify significance.
(1.01 MB XLS)

Table S7 Peripheral endothelial enriched genes. A data set containing probe sets from the mouse 430–2 GeneChip statistically enriched in peripheral endothelial cells compared with those from the CNS as identified by probe sets with LiverGFP^+^:BrainGFP^+^ >2 and LungGFP^+^:BrainGFP^+^ >2 with expression value in LiverGFP^+^>200 and LungGFP^+^>200. SAM algorithms were utilized to identify significance.
(0.28 MB XLS)
